# The hemodynamic cost of intermittent hemodialysis and systemic organ stunning 

**DOI:** 10.1080/0886022X.2026.2727734

**Published:** 2026-09-21

**Authors:** Zongchen Jiang, Linjie Zhong, Jungang Fang, Chang Liu, Ran Ji, Yange Hu, Xin Zhao, Wensheng Qi

**Affiliations:** ^a^ Guang’anmen Hospital, China Academy of Chinese Medical Sciences, Beijing, China; ^b^ Graduate School, Beijing University of Chinese medicine, Beijing, China; ^c^ Department of Emergency, Guang’anmen Hospital, Beijing, China 5 Beixian’ge Street, Xicheng District

**Keywords:** Intermittent hemodialysis, repetitive dialytic stress syndrome, ischemia-reperfusion injury, myocardial stunning, intradialytic hypotension, hemodynamic shear stress

## Abstract

Despite advancements in solute clearance technologies, cardiovascular mortality in maintenance intermittent hemodialysis (IHD) patients remains disproportionately high. The traditional clearance-based paradigm fails to fully explain this survival gap. Here, we propose conceptualizing IHD as a repetitive iatrogenic stressor and introduce the framework of Repetitive Dialytic Stress Syndrome (RDSS). We postulate that rapid fluctuations in intravascular volume and osmolarity impose a cyclical hemodynamic burden, inducing subclinical microcirculatory shock. Current evidence demonstrates how this recurrent perfusion deficit triggers systemic ischemia-reperfusion injury. This cascade manifests as synchronous organ stunning, driving myocardial fibrosis, cerebral white matter injury, gut endotoxemia, and residual kidney function loss. By redefining dialysis adequacy to include hemodynamic stability, we highlight how therapeutic strategies optimizing physiological stability can mitigate RDSS and improve long-term outcomes.

## LAY (PLAIN LANGUAGE) SUMMARY

Hemodialysis is a life-saving treatment for kidney failure, but cardiovascular mortality in this population remains unusually high. While dialysis successfully clears toxins, the rapid removal of excess body water acts as a repetitive physical shock. We propose the Repetitive Dialytic Stress Syndrome (RDSS) concept to describe how these cyclical shocks cause temporary drops in blood flow to the heart, brain, and gut, ultimately leading to chronic inflammation and irreversible organ damage. Understanding dialysis as a physical stress test enables clinicians to use personalized strategies to maintain hemodynamic stability, protect vital organs, and improve survival.

## 1. Introduction

Despite decades of technical refinement in maintenance hemodialysis (MHD), the long-term prognosis for patients with end-stage kidney disease remains stark. Cardiovascular mortality in this population exceeds that of the general population by 10- to 20-fold and persists significantly higher than that of patients managed with peritoneal dialysis or kidney transplantation [1–3]. Historically, nephrology has operated under a ‘toxin-centric’ paradigm, attributing this excess risk primarily to the retention of uremic solutes and chronic inflammation [4,5]. Consequently, the therapeutic imperative has focused on maximizing solute clearance (e.g., Kt/V), often at the expense of hemodynamic stability. However, the failure of intensified clearance trials to consistently yield survival benefits suggests that uremic toxicity alone cannot fully account for the observed systemic pathology [6,7]. This discrepancy points to a critical, overlooked pathogenic mechanism inherent to the treatment modality itself: the profound hemodynamic cost of the extracorporeal circuit, where the pursuit of biochemical purity inadvertently compromises systemic perfusion.

**Figure 1.**
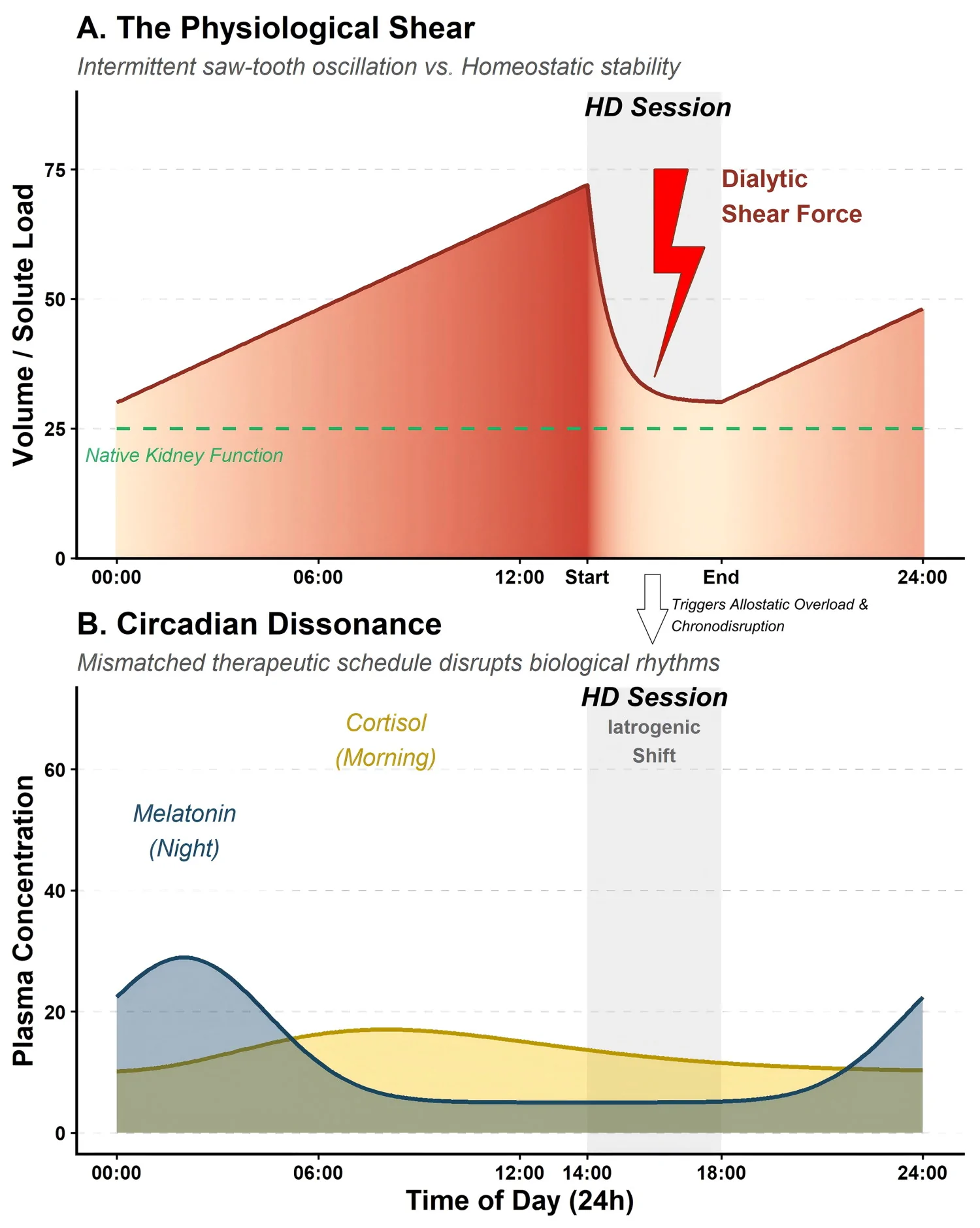
The Physiological Dissonance of Intermittent Hemodialysis.

From a critical care perspective, the standard intermitted hemodialysis (IHD) session represents more than a clearance procedure; it acts as a repetitive physiological stress test. By compressing the homeostatic regulation native kidneys achieve in 168 h into an aggressively compressed 12-h weekly window, IHD subjects the patient to rapid, supra-physiological shifts in intravascular volume and metabolic composition (Figure 1(A)). While recent reviews have elucidated specific pathways of dialysis-induced injury in the brain [8] and heart [9], focusing on isolated organs obscures the broader mechanobiological reality. We propose that these organ-specific injuries are localized manifestations of a systemic failure in microcirculatory perfusion. We identify the sinusoidal oscillation of the dialysis cycle not merely as a hemodynamic event, but as a repetitive biophysical perturbation – a ‘subclinical shock’ state – that disrupts cellular homeostasis at the molecular level.

In this review, we introduce the ‘Hemodynamic Cost’ hypothesis. We postulate that the IHD procedure generates a spectrum of damaging physical gradients – ranging from turbulent shear stress at the vascular access to osmotic shifts in the microcirculation – which trigger a pathological cascade of aberrant mechanotransduction. By analogy to the mechanical shear stress exerted by fluid flow on the vascular endothelium, we expand this concept to include the rapid osmotic and metabolic fluxes that act as physical signals detected by cellular mechanosensors. This review integrates evidence exploring how this cyclical biophysical insult could theoretically be transduced into a continuous cascade of repetitive, sub-lethal hypoxic strain, mitochondrial dysfunction, and sterile inflammation across the heart, brain, and gut [10,11]. Importantly, to transition RDSS from a conceptual hypothesis to a clinically actionable entity, we propose a preliminary set of operational criteria based on recent hemodynamic and molecular evidence (Table 1). By establishing specific clinical triggers (e.g., absolute nadir blood pressure) and corresponding organ phenotypes, we aim to differentiate routine dialytic fatigue from pathological mechanotransduction. This pathogenic cascade begins at the most fundamental level of fluid mechanics: the interplay between the extracorporeal circuit and systemic perfusion.

**Table 1.** Proposed operational framework and diagnostic criteria for RDSS.

Domain	Proposed marker/diagnostic threshold	Rationale & clinical evidence
Clinical/Hemodynamic triggers	Absolute Nadir SBP <90 mmHg (or<100 mmHg if pre-HD SBP ≥160) [12–14]. Ultrafiltration Rate > 13 mL/h/kg.	Absolute nadir achieved is more predictive of 5-year mortality than relative BP decline. High UFR drives systemic perfusion-metabolism mismatch.
Cardiac phenotype (myocardial stunning)	STE : Development of ≥2 Regional Wall Motion Abnormalities (RWMAs) [15,16]. GLS: Acute worsening of Global Longitudinal Strain by ≥20% from baseline.	Correlates temporally with peak intradialytic stress; strongly predicts progression to fixed systolic dysfunction and mortality.
Neurological phenotype (cerebral stunning)	Acute: Increased Fractional Anisotropy (FA) and decreased Mean Diffusivity (MD) on DTI [17]. Chronic: Progression of White Matter Hyperintensities (Fazekas scale ≥2) [18].	Acute DTI changes reflect cytotoxic edema during HD. Cumulative burden manifests as progressive leukoaraiosis in watershed zones.
Molecular / inflammatory signatures	IL-6 Spike: >50–60% increase above baseline at 2 h post-dialysis [19,20]. Transient Endotoxemia: Detected with (1→3)-β-D-glucan blocking buffers.	Interleukin-6 represents a reliable, acute-phase readout of intradialytic bioincompatibility and sub-clinical ischemic stress.

**Note:** hs-cTnT typically decreases immediately post-dialysis due to dialytic clearance, thus acute intradialytic changes are unreliable for immediate stunning detection without correcting for hemoconcentration [21,22]. STE, speckle-tracking echocardiography; DTI, diffusion tensor imaging

## 2. The hemodynamic insult

The pathogenesis of RDSS is fundamentally rooted in the violation of biophysical laws. The mammalian cardiovascular system is evolutionarily optimized to manage pulsatile, laminar flow within a tightly regulated pressure range, ensuring continuous microcirculatory perfusion. IHD challenges these biophysical principles by imposing a supra-physiological mechanical load that acts as a primary signaling event. We categorize this biophysical perturbation into two distinct mechanotransduction inputs: the micro-environmental turbulence at the vascular interface and the macro-environmental instability of systemic perfusion.

### 2.1. Vascular shear stress

The arteriovenous fistula (AVF) of renal dialysis functions not merely as a passive mechanical conduit for blood removal but as an upstream generator of pathological molecular signals. It creates a low-resistance, high-flow shunt where fluid velocities exceed physiological norms by an order of magnitude. Unlike the uniform, laminar shear stress that promotes endothelial homeostasis *via* eNOS activation and nitric oxide (NO) production, the flow dynamics at the AVF anastomosis – specifically within the venous outflow tract – are characterized by high-frequency turbulence and a pathologically elevated oscillatory shear index [23,24].

This aberrant physical force is detected by specific endothelial mechanosensors, most notably the Piezo1 ion channels and the fragile endothelial glycocalyx. We hypothesize that this ‘reciprocating’ shear stress triggers a phenotypic switch in endothelial cells. The mechanical deformation of the cytoskeleton activates downstream signaling pathways, upregulating pro-inflammatory cytokines (e.g., MCP-1, IL-8) and promoting smooth muscle cell proliferation [25,26]. Crucially, this pathology disseminates systemically. The mechanically activated endothelium sheds endothelial microparticles and oxidative byproducts into the central circulation. These circulating vectors effectively ‘prime’ the distal vasculature, converting a localized biophysical stress into a systemic inflammatory signal [27,28].

### 2.2. Systemic perfusion mismatch

While flow disturbances at the vascular access represent a localized mechanotransduction event, the dialysis session itself imposes a systemic hemodynamic burden best described as a perfusion-metabolism mismatch. In contrast to the homeostatic autoregulation of the native kidney, the extracorporeal circuit enforces a rigid ultrafiltration rate (UFR) that frequently exceeds the plasma refill rate of the vascular compartment [29].

**Figure 2.**
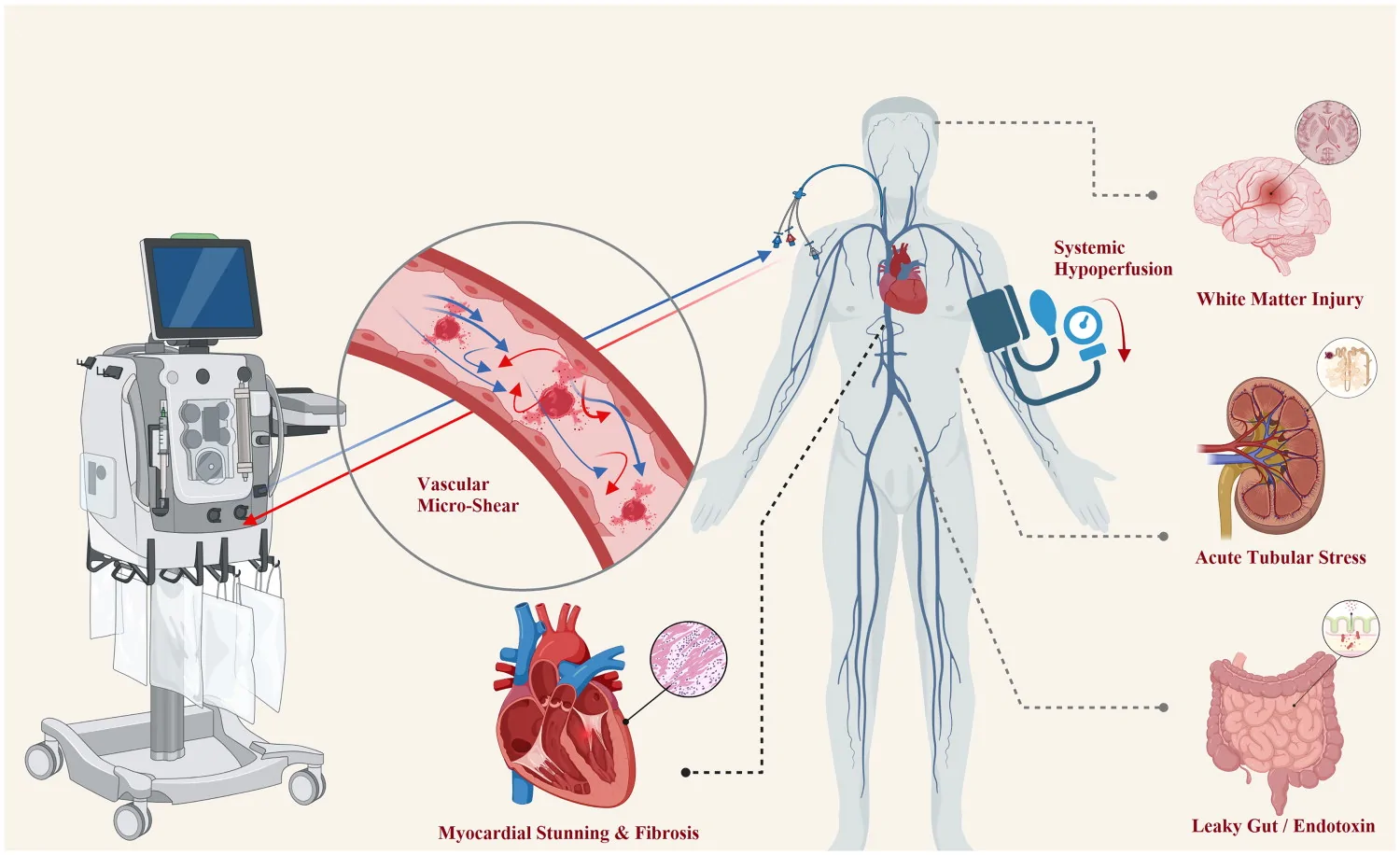
From Hemodynamic Insult to Multi-Organ Stunning.

Data demonstrate that high UFRs (>10–13 mL/h/kg) function as an independent biophysical stressor, uncoupling systemic pressure from tissue perfusion. This creates a state of ‘Occult Shock’ or ‘Subclinical Ischemia’ (Figure 2), where the oxygen delivery (DO_2_) falls below the critical threshold required for mitochondrial respiration, even before overt arterial hypotension is detected [30,31]. Observations of elevated cardiac biomarkers (troponin I, CK-MB) following asymptomatic pressure dips provide direct evidence that routine hemodialysis induces episodes of subclinical myocyte necrosis akin to silent ischemia [32,33]. This creates a maladaptive feedback loop: chronic interdialytic volume overload (‘the peak’) drives left ventricular hypertrophy and fibrosis, rendering the heart stiff [34,35]. This compromised myocardium is then subjected to the acute preload withdrawal of the subsequent dialysis session (‘the trough’). Thus, the macro-insult of IHD acts as a cyclical driver of structural remodeling, wherein hemodynamic fluctuations function as potent biophysical determinants of cell fate [36,37].

## 3. The mechanotransduction cascade

**Figure 3.**
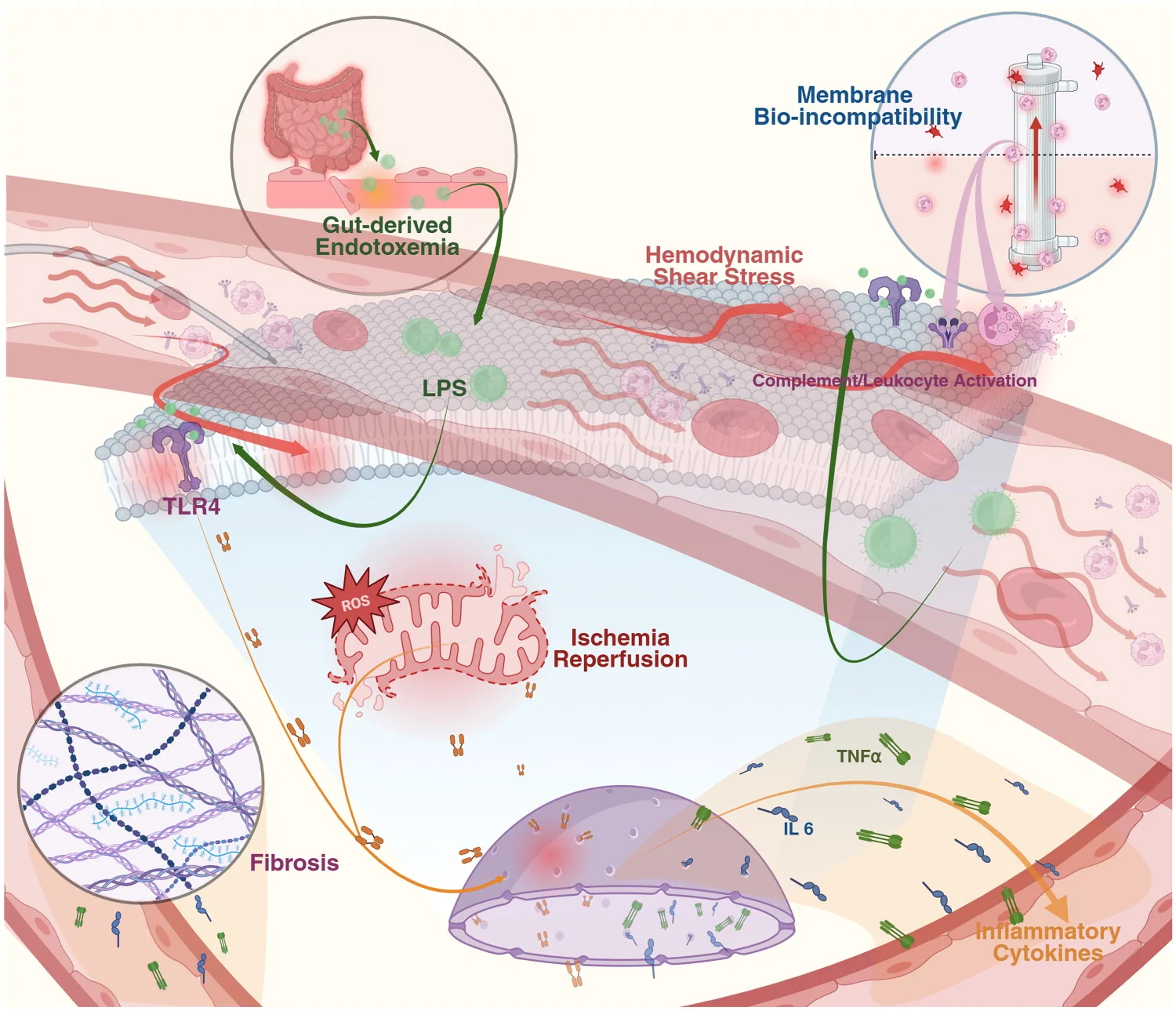
The Molecular Transduction: From Hemodynamic Stress to Sterile Inflammation.

While the biophysical insults described above provide the physical ‘trigger’, the progression to chronic systemic pathology requires a molecular transducer to convert mechanical stress into biological toxicity. Although specific mechanosensors such as the endothelial glycocalyx or ion channels likely participate in sensing these shear forces, the dominant pathological driver is a systemic ‘Two-Hit’ molecular cascade. We hypothesize that the IHD session phenotypically induces a state of repetitive, sub-lethal hypoxic strain – distinct from acute, catastrophic ischemia-reperfusion injury (IRI) – which is subsequently amplified by a gut-derived inflammatory axis (Figure 3).

### 3.1. Mitochondrial uncoupling

The obligate periodicity of IHD imposes a unique metabolic rhythm: cyclical hypoxia followed by abrupt re-oxygenation. During the peak ultrafiltration phase, microcirculatory rarefaction compromises oxygen delivery to high-demand tissues (myocardium, brain cortex, proximal tubules), inducing regional ATP depletion [38,39]. This transient ischemic phase primes the mitochondrial electron transport chain for dysfunction. The subsequent ‘reperfusion’ event – occurring as vascular volume is restored or intradialytic vascular tone relaxes – triggers electron leakage at Complex I and III, generating aberrant bursts of reactive oxygen species (ROS), primarily superoxide anions (O2•−) and hydrogen peroxide (H2O2) [40–42].

This oxidative burden is critically exacerbated by a failure in mitochondrial quality control. Protein-bound uremic toxins, such as indoxyl sulfate, potently block autophagic flux and disrupt PINK1/Parkin-mediated mitophagy [43]. Consequently, damaged, uncoupled mitochondria are not efficiently cleared but instead accumulate intracellularly. Unlike discrete ischemic events, the cyclical nature of dialysis not only exhausts the patient’s antioxidant reserves (e.g., glutathione) but creates a vicious cycle where these accumulated dysfunctional mitochondria act as perpetual ROS generators. This systemic failure in mitophagy locks the cellular environment in a permanent phenotype of ‘Cyclical Oxidative Instability’ [44,45] and promotes downstream apoptosis [46,47]. This chronic oxidative stress damages mitochondrial DNA (mtDNA) and triggers the opening of the mitochondrial permeability transition pore (mPTP), shifting cellular signaling from metabolic adaptation to pro-apoptotic cascades.

### 3.2. The gut-vascular axis

This intrinsic oxidative burden is synergistically amplified by a specific iatrogenic pathway: the gut-systemic inflammatory axis. Dynamic CT and regional blood flow studies demonstrate that IHD induces a transient reduction in splanchnic perfusion (e.g., decreasing from ∼0.9 to 0.7 L/m^2^/min), which recovers post-dialysis [48–50]. Rather than the catastrophic gut leak seen in critical care sepsis, this recurrent mild-to-moderate ischemic strain is hypothesized to cause transient mucosal permeability alterations. Prolonged exposure to intradialytic hypotension progressively depletes tight junction proteins (e.g., claudin-1, occludin) [49,51]. Consequently, this compromised barrier permits the episodic translocation of luminal endotoxin (lipopolysaccharide, LPS) [52,53]. It is noteworthy, however, that the exact magnitude of intradialytic endotoxemia remains debated, as recent evidence suggests standard limulus amebocyte lysate assays may yield false positives due to (1→3)-β-D-glucan interference in uremic patients [54–57]. Nonetheless, the downstream inflammatory response is robustly validated, with acute phase cytokines like IL-6 surging up to 68% above baseline within two hours post-dialysis [58]. Circulating LPS acts as a potent ligand for Toll-like receptor 4 on endothelial and immune cells. Upon binding, TLR4 recruits MyD88 and activates the NF-κB signaling cascade, driving the transcription of pro-inflammatory cytokines (TNF-α, IL-6) [52,58]. Simultaneously, blood contact with the extracorporeal membrane triggers the complement cascade (C3a/C5a) and promotes neutrophil extracellular trap formation [59,60].

Consequently, these convergent pathways establish a unified molecular mechanism of injury: the uremic milieu provides the background toxicity that primes the endothelium, while the repetitive hemodynamic (biophysical) and reperfusion (metabolic) insults of the treatment session act as the recurrent trigger. This ‘Mechanotransduction-Inflammation Axis’ drives the transcriptional reprogramming of vascular and parenchymal cells toward a fibrotic phenotype, ultimately leading to end-organ failure [51,61–64].

## 4. Systemic organ stunning

From a critical care perspective, systemic arterial pressure is often a poor surrogate for microcirculatory adequacy. While intense compensatory vasoconstriction may preserve mean arterial pressure, it masks a critical molecular reality: the sequential sacrifice of end-organ bioenergetics. We propose that the clinical phenomenon of ‘stunning’ (Figure 2) – originally described as a mechanical dysfunction – represents a ubiquitous state of metabolic supply-demand mismatch affecting the myocardium, brain, gut, and residual kidney [65–67].

### 4.1. Myocardial hibernation

The myocardium serves as the sentinel tissue for repetitive perfusion injury. While intradialytic H_2_^15^O positron emission tomography has clinically validated the presence of regional wall motion abnormalities independent of epicardial coronary disease [68], the underlying pathology is molecular in nature. This ‘myocardial stunning’ represents a state of acute bioenergetic failure driven by a critical mismatch between oxygen supply (reduced coronary perfusion pressure due to ultrafiltration) and metabolic demand (sympathetic surge-driven contractility).

Unlike the acute, irreversible necrosis characteristic of myocardial infarction, this recurrent sub-clinical ischemia initially drives an adaptive response akin to myocardial hibernation, downregulating contractile function to preserve cell viability [69–72]. However, this compensatory metabolic remodeling is ultimately undermined by the uremic milieu. Chronic kidney disease induces severe capillary rarefaction and a paradoxically blunted hypoxia-inducible factor (HIF-1α) response, depriving the myocardium of essential angiogenic adaptations. Consequently, the resulting leakage of ROS and pro-apoptotic factors from uncoupled mitochondria initiates a maladaptive repair process. Recent 3D speckle tracking data confirms that this mechanical strain is not benign but cumulative, activating profibrotic signaling pathways (e.g., TGF-β) that drive the transition from reversible myocyte dysfunction to fixed interstitial fibrosis and cardiomyocyte death [73–75]. Thus, uremic cardiomyopathy should be reconceptualized not merely as a volume-overload state, but as a disease of cumulative, demand-driven ischemic remodeling.

### 4.2. Cerebral white matter injury

The vulnerability of the cerebrovascular bed to dialytic stress highlights the fragility of the neurovascular unit. Chronic hypertension and arteriosclerosis shift the brain’s autoregulatory curve to the right, rendering cerebral tissue susceptible to hypoperfusion even at ‘safe’ MAP levels. Molecularly, this manifests as a disruption of the blood-brain barrier and cytotoxic edema [9].

Advanced neuroimaging provides structural evidence of this molecular trauma. Longitudinal diffusion tensor imaging reveals a specific degradation of white matter integrity, characterized by ‘pothole-like’ microstructural defects [76,77]. White matter, rich in lipid-heavy myelin, is particularly vulnerable to the oxidative stress generated during the reperfusion phase of dialysis. Furthermore, the rapid osmotic shifts inherent to IHD induce transient cerebral edema, mechanically compressing the microcirculation and exacerbating local hypoxia. This mechanism underpins the ‘vascular dementia’ phenotype observed in dialysis patients, driven by repetitive ‘watershed’ ischemia and the accumulation of silent lacunar infarcts [78–80]. Essentially, ‘Cerebral Stunning’ is the clinical correlate of progressive neuro-axonal degeneration [81,82].

### 4.3. Tubular senescence

For incident dialysis patients, the preservation of residual kidney function (RKF) is a critical survival determinant. However, aggressive IHD accelerates RKF decline *via* the same ischemic mechanisms affecting other organs [83,84]. Intradialytic renal hypoperfusion imposes a repetitive ‘acute-on-chronic’ ischemic insult to the proximal tubules. At the molecular level, the cellular response to hypoxia in the kidney sharply contrasts with that of the myocardium. While the uremic myocardium exhibits a paradoxically blunted HIF-1α response due to uremic toxin-mediated transcriptional suppression (e.g., *via* CITED2 and AhR activation) and epigenetic hypermethylation of angiogenic genes [85,86], the renal proximal tubule demonstrates divergent, sustained HIF-1α accumulation. In the kidney, heavy oxidative stress and proteinuria directly inhibit prolyl hydroxylase activity, preventing HIF-1α degradation [87]. Rather than promoting adaptive angiogenesis, this aberrant, sustained HIF-1α signaling directly binds the hypoxia-response element on the p53 promoter [88], forcing tubular epithelial cells into a prolonged G2/M cell cycle arrest [88]. Rather than undergoing normal repair, these G2/M-arrested cells adopt a chronic senescence-associated secretory phenotype, releasing a continuous barrage of profibrotic mediators (e.g., TGF-β, IL-6) into the interstitium [89,90]. This mechanotransduction pathway converts reversible tubular strain into progressive tubulointerstitial fibrosis, accelerating the irreversible loss of functional nephrons.

## 5. Circadian desynchronization

Beyond the overt perturbations of perfusion pressure and tissue architecture lies a profound disruption of the organism’s temporal homeostasis: the violation of biological time at the transcriptional level (Figure 1(B)). Physiological processes – from mitochondrial bioenergetics to DNA repair and vascular tone – are orchestrated by intrinsic circadian clocks driven by the transcription-translation feedback loop involving core clock genes such as BMAL1, CLOCK, PER, and CRY. Under normal physiological conditions, these peripheral clocks (in the kidney, heart, and vasculature) are synchronized by the ‘Master Clock’ in the suprachiasmatic nucleus. However, standard Intermittent Hemodialysis (IHD) functions as a potent, non-photic ‘Zeitgeber’ (time-giver) that forces an abrupt, extrinsic disruption of this internal timekeeping, creating a state of molecular ‘Chronodisruption.’

The standard IHD schedule imposes rapid, supra-physiological shifts in body temperature, solute concentrations, and volume status that are often diametrically opposed to the patient’s intrinsic circadian phase [91]. This creates a severe internal desynchronization between the central SCN clock and peripheral tissue clocks. For instance, the rapid clearance of small molecules (some of which act as metabolic signaling intermediates) and the sudden induction of hypothermia (*via* dialysate cooling) can reset peripheral clock gene expression in a chaotic manner. Even in the absence of uremia, the loss of renal function blunts the nocturnal ‘dipping’ of blood pressure and disrupts melatonin signaling. The IHD procedure exacerbates this by preventing the restorative physiological ‘downtime’ essential for cardiovascular repair and the re-synthesis of antioxidant enzymes [92,93]. This temporal misalignment drives the neuro-endocrine axis into a state of chronic allostatic overload. To defend arterial pressure against the aggressive fluid removal of IHD, the sympathetic nervous system and the renin-angiotensin-aldosterone system are recruited to their physiological limits. At the molecular level, this results in a continuous, high-amplitude adrenergic signaling bombardment on cardiomyocytes and endothelial cells. This chronic G-protein coupled receptor activation leads to receptor desensitization (downregulation of β-adrenergic receptors) and alters intracellular Ca^2+^ handling. The clinical manifestation of ‘Autonomic Dysfunction’ or ‘Blunted Heart Rate Variability’ is, in essence, the macroscopic readout of this exhausted molecular regulatory machinery [94,95]. The RDSS framework thus posits that chronodisruption constructs a pro-arrhythmic substrate: a fibrotic myocardium with ion channel remodeling, sensitized by rapid electrolyte flux and deprived of vagal protection [96,97].

## 6. Preserving microcirculatory homeostasis

Validating the mechanotransduction hypothesis requires demonstrating that therapeutic interventions targeting these biophysical forces can successfully mitigate end-organ injury (Table 2). Rather than viewing dialysis adequacy exclusively through the lens of solute kinetics (Kt/V), we must reprioritize interventions that dampen specific nodes of the RDSS cascade. The most direct method to dismantle this aberrant mechanotransduction is to blunt the macroscopic ‘peak-to-trough’ variance of the treatment cycle itself. Applying the RDSS framework clinically requires strict patient stratification to resolve seemingly contradictory therapeutic approaches. For prevalent, anuric patients prone to severe volume overload and IDH, strategies that intensify treatment frequency (such as daily or nocturnal hemodialysis) are indicated. These regimens effectively flatten the ‘peak-to-trough’ variance, reducing the hourly ultrafiltration rate and dampening the physical shear stress detected by the endothelial glycocalyx, consistently demonstrating regression of left ventricular hypertrophy [100,114]. Conversely, for incident patients with robust RKF, an incremental hemodialysis approach (e.g., twice-weekly) is preferred. In this phenotype, deliberately minimizing extracorporeal circuit exposure functions to protect fragile tubular architecture from recurrent hypoxic strain, thereby preserving RKF [98,99]. Furthermore, addressing chronodisruption necessitates chronotherapy – adjusting dialysis shift timing to avoid peak ultrafiltration during periods of physiological vulnerability (e.g., nocturnal blood pressure dipping phases), thus aligning the mechanical stress with the patient’s intrinsic circadian tolerance.

**Table 2.** The toolbox of physiological preservation: Patient-Stratified strategies to mitigate RDSS.

Therapeutic strategy	Target node	Physiological & molecular mechanism	Eligible patient phenotype (stratification)	Supporting evidence
Incremental Hemodialysis (e.g., 2x/week initiation)	Extracorporeal Circuit Exposure (Preserving residual function)	**Graded Stress:** Reduces initial cumulative exposure to bioincompatible surfaces and limits ‘dialysis shock’ to residual nephrons.	**Incident dialysis patients** with robust residual kidney function (urine output >600 mL/day or renal urea clearance >3 mL/min) and no volume overload.	Meta-analyses show superior preservation of RKF compared to standard 3x/week start [98,99].
Intensified Regimens (Frequent Daily or Nocturnal HD)	Hemodynamic Volatility & Chronodisruption (Minimizing peak-trough amplitude)	**Flattening the Curve:** Drastically reduces interdialytic volume accumulation and UFR. **Chronobiological Restoration:** Nocturnal HD aligns treatment with physiological sleep and melatonin peaks.	**Prevalent, anuric patients** complicated by severe volume overload, frequent IDH, therapy-resistant hypertension, or sleep disorders.	RCT (FHN Trial) & Meta-analyses: Regression of LV hypertrophy, improved BP dipping, and sleep parameters [100–105].
Dialysate Cooling (0.5 °C below core temp)	Metabolic Supply-Demand Mismatch Preventing sub-lethal ischemia)	**Bioenergetic Conservation:** Promotes physiologic venoconstriction to sustain central perfusion pressure and macroscopic hemodynamics while lowering local tissue metabolic demand.	**Hypotension-prone patients**; patients with documented intradialytic myocardial stunning (*via* STE) or established ischemic cardiomyopathy.	Meta-analyses show reduced frequency of IDH and mitigation of myocardial stunning [106–108].
High-Volume HDF (Convection >23 L)	Sterile Inflammation Improving molecular environment)	**Enhanced Convection:** Efficiently clears middle-molecular-weight pro-inflammatory cytokines, reducing the baseline inflammatory background.	**Long-term dialysis patients** (>10 years) at high risk for cardiovascular mortality and β 2-microglobulin amyloidosis.	Large RCTs (e.g., CONVINCE) and pooled analyses show reduced all-cause/CV mortality vs. high-flux HD [109].
Intradialytic Exercise (Aerobic/Resistance)	Endothelial Dysfunction (Preconditioning the vasculature)	**Ischemic Preconditioning:** Generates physiologic laminar shear stress to counteract AVF-induced turbulence; upregulates eNOS.	**Hemodynamically stable patients** with sarcopenia or reduced CV reserve; strictly contraindicated in uncontrolled arrhythmias/hypertension.	RCTs demonstrate acute mitigation of myocardial stunning and improved arterial stiffness [110–112].
Intradialytic EECP	Venous Return & Preload (Counteracting hypovolemia)	**Hemodynamic Augmentation:** Diastolic compression actively increases cardiac preload and coronary perfusion pressure, maintaining cardiac output. **Endothelial Conditioning:** Enhances physiological shear stress.	**Hemodynamically unstable patients** prone to subclinical cardiac output decline; contraindicated in severe peripheral arterial disease or decompensated heart failure.	Preliminary RCT (2025) shows preservation of cardiac output and potential attenuation of myocardial injury markers (hs-cTnI) during HDF [113].

**Note:** BP, blood pressure; CV, cardiovascular; HDF, hemodiafiltration; IDH, intradialytic hypotension; LV, left ventricular; MAP, mean arterial pressure; RCT, randomized controlled trial; RKF, residual kidney function; EECP, Enhanced External Counterpulsation; STE, speckle-tracking echocardiography; eNOS, endothelial nitric oxide synthase.

Beyond altering the treatment schedule, preserving microcirculatory homeostasis requires active hemodynamic modulation to match metabolic supply and demand during the intradialytic phase. Drawing from critical care principles of shock management, preventing ischemic injury involves not only sustaining perfusion pressure but also minimizing tissue oxygen consumption (VO_2_). Standard dialysis frequently induces thermal stress and inappropriate vasodilation, precipitating a systemic supply-demand mismatch. The application of individualized cooled dialysate (e.g., 0.5 °C below core temperature) directly counters this by acting as a metabolic brake. It promotes compensatory venoconstriction to sustain mean arterial pressure while simultaneously lowering the metabolic requirements of vulnerable capillary beds. This thermodynamic protection preserves mitochondrial integrity during the aggressive ultrafiltration phase, profoundly abrogating regional myocardial stunning and facilitating structural reverse remodeling [107,108].

Furthermore, because the physical stress of IHD is synergistically amplified by a toxic biochemical milieu, comprehensive protection necessitates modulating the molecular environment and enforcing endothelial resilience. High-volume hemodiafiltration utilizes convective forces to clear middle-molecular-weight uremic toxins and oxidative byproducts that diffuse poorly in standard hemodialysis. As these molecules serve as priming ligands for NF-κB activation, reducing the circulating inflammatory burden raises the endothelial threshold for shear-induced injury, thereby interrupting the ‘sterile inflammation’ feedback loop [110,111,115]. Finally, the vasculature can be actively conditioned to withstand dialytic stress through intradialytic exercise, which functions as a form of non-pharmacological ‘ischemic preconditioning’. Unlike the pathological, oscillatory shear stress generated at the arteriovenous fistula, exercise induces physiological, laminar shear stress. This active recruitment stimulates endothelial nitric oxide synthase phosphorylation and upregulates endogenous antioxidant systems. Randomized data confirming that intradialytic exercise can acutely reverse regional wall motion abnormalities provide definitive proof that the myocardium remains salvageable when protective, physiological signaling pathways are engaged to counteract the iatrogenic stress of dialysis [112].

Beyond pharmacological or schedule-based modifications, emerging mechanical assist devices offer a direct biophysical countermeasure against RDSS. Recently, the application of EECP during hemodialysis has demonstrated physiological promise. By sequentially compressing the lower-extremity vascular beds during diastole and rapidly deflating during systole, EECP actively augments venous return and cardiac preload, effectively counteracting the hypovolemia induced by rapid ultrafiltration [113,116]. A recent preliminary randomized controlled trial (2025) revealed that a 60-min concurrent EECP session successfully preserved cardiac output throughout hemodiafiltration and showed a trend toward attenuating high-sensitivity cardiac troponin I elevation, suggesting acute mitigation of myocardial ischemic strain [113]. Furthermore, from a mechanotransduction perspective, the pulsatile flow generated by EECP upregulates endothelial nitric oxide synthase (eNOS) *via* physiological shear stress, promoting systemic endothelial conditioning [117,118]. While larger trials are required to determine its impact on hard clinical endpoints and to evaluate contraindications in the complex ESRD population, EECP exemplifies how manipulating macroscopic hemodynamics can theoretically intercept the pathological mechanotransduction of dialytic organ stunning.

## 7. Limitations, contradictory evidence, and the necessity of patient stratification

While the RDSS hypothesis provides a cohesive mechanotransduction framework connecting intermittent hemodynamic stress to multi-organ stunning, translating this paradigm into universal clinical practice remains challenging. A critical limitation of current literature is the reliance on surrogate markers (e.g., global longitudinal strain, circulating endotoxin, or white matter hyperintensities) rather than hard clinical endpoints, making it difficult to fully disentangle RDSS from the overlapping effects of chronic uremia, aging, and preexisting vascular comorbidities.

Furthermore, interventions designed to mitigate dialytic stress (as outlined in Table 1) do not uniformly improve survival and carry distinct therapeutic tradeoffs. The pursuit of absolute hemodynamic stability through intensified hemodialysis (frequent or prolonged sessions) is a prime example of this paradox. While hemodynamically sound, the Frequent Hemodialysis Network (FHN) trial and subsequent analyses demonstrated that intensified regimens significantly increase the burden of vascular access complications and infections [83]. Moreover, in incident patients, frequent HD can ironically accelerate the loss of residual kidney function [119], highlighting why an incremental approach is preferred for the incident phenotype to minimize circuit exposure [120,121]. Thus, prescribing intensive versus incremental HD represents a calculated tradeoff between volume control and circuit exposure, demanding strict patient stratification (Table 1) rather than a one-size-fits-all protocol.

Similarly, mitigating metabolic supply-demand mismatch *via* dialysate cooling – a widely recommended strategy to prevent IDH – has yielded contradictory results in recent rigorously designed studies. A recent randomized controlled trial by Gullapudi et al. (2024) challenged the end-organ protective effects of thermocontrolled hemodialysis, demonstrating no definitive superiority over standard dialysis in protecting the heart, brain, or kidneys assessed *via* multiparametric MRI [122]. Furthermore, cooling often reduces patient body temperature to poorly tolerated levels, compromising patient adherence and comfort [123].

Even intradialytic exercise, which theoretically provides ischemic preconditioning and physiological laminar shear stress to counteract endothelial dysfunction, carries risks. While generally deemed safe [111], it can provoke severe cardiovascular events and remains absolutely contraindicated in patients with severe hypertension (>180/110 mmHg) or uncontrolled arrhythmias [124]. Finally, the survival benefit of high-volume HDF continues to be debated in specific subpopulations, as optimal convection volumes are difficult to achieve in routine clinical practice without reliable vascular access [109].

In summary, the RDSS hypothesis does not advocate for a single ‘perfect’ dialysis prescription. Rather, it emphasizes that dialytic stress is heterogeneous. Recognizing the contradictory clinical evidence underscores the necessity of personalized hemodialysis – deploying specific interventions based on whether a patient’s dominant RDSS phenotype is driven by ultrafiltration volatility, circuit bioincompatibility, or chronodisruption.

## 8. Conclusion

The intractable mortality burden in end-stage kidney disease suggests that current pharmacological and technological interventions overlook a fundamental pathogenic driver. We posit that the ‘missing link’ lies in the biophysical nature of the therapy itself. Intermittent hemodialysis should not be viewed merely as a solute-clearance technique, but as a repetitive biophysical perturbation that imposes a cumulative mechanotransduction load on cellular homeostasis.

Through the hypothesis of RDSS, we integrate fragmented observations of organ injury into a unified molecular framework. We propose that the macroscopic forces of hemodynamic shear and osmotic flux act as upstream signaling events, which are sensed by the endothelium and transduced into intracellular cascades of mitochondrial dysfunction, oxidative stress, and sterile inflammation. Thus, the clinical phenotypes of myocardial stunning, cerebral white matter injury, and gut translocation are not isolated complications, but synchronized molecular sequelae of the same aberrant physical signaling. This paradigm shift compels a reevaluation of dialysis adequacy beyond the kinetics of small-molecule clearance. Future research must focus on deciphering the precise molecular sensors (e.g., mechanosensitive ion channels, integrins) that convert dialytic stress into biological toxicity. Ultimately, bridging the survival gap requires a transition from simply ‘cleaning the blood’ to restoring molecular and bioenergetic homeostasis, potentially *via* therapeutic strategies that dampen these pathological mechanotransduction pathways.

## Data Availability

Data sharing is not applicable to this article as no new data were created or analyzed in this study.
